# Insights from the International Registry of Acute Aortic Dissection

**DOI:** 10.21542/gcsp.2016.8

**Published:** 2016-03-31

**Authors:** Arturo Evangelista, Giuliana Maldonado, Doménico Gruosso, Gisela Teixido, Jose Rodríguez-Palomares, Kim Eagle

**Affiliations:** 1 Servei de Cardiología. Hospital Universitari Vall d’Hebron. Barcelona; 2 Cardiovascular Center. University of Michigan. Ann Arbor

## Introduction

**Figure 1.**
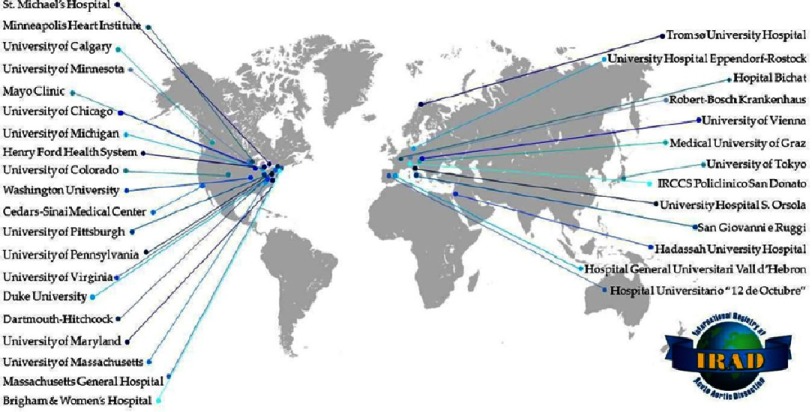
IRAD centres worldwide. IRAD. International Registry Acute Aortic Dissection. Reproduced with permission (IRAD Center?).

The International Registry of Acute Aortic Dissection (IRAD) was established in 1996 for the purpose of enrolling patients at large referral centres to assess the presentation, management and outcomes of acute aortic dissection (AAD). Data on presentation, diagnostic, management and outcomes were initially collected by 12 centres and then extended to 28 referral centres (Figure 1). All data of more than 5,000 cases were reviewed and analysed by the IRAD Coordinating Center at the University of Michigan^1^. Since the first publication in 2000, IRAD investigators have reported a number of clinical observations, in more than 70 publications. This article will cover most of these points highlighting the findings of IRAD in patients with type A (with ascending aorta involvement) and type B (without ascending aorta involvement) AAD.

## Risk factors

In the IRAD series, two thirds of included patients were male, with a mean age for all patients of 63 years. Women were older than men, with a mean age of 67 years (p=0.008)^2^. There are many risk conditions related to aortic dissection. The most common predisposing factor in the IRAD was hypertension (72%). In the total registry, 5% and 4% of AAD were thought to be related to Marfan’s syndrome and iatrogenic causes, respectively^3^ and cocaine use in 1.8%^4^. Younger patients (< 40 years of age) were less likely to have a history of hypertension (34%) or atherosclerosis (1%) but more likely to have Marfan’s syndrome and bicuspid aortic valve (59 %)^5^. A history of atherosclerosis was present in 31% of patients and a history of heart surgery in 18%^6^. Although aorta dilation is a well-established risk factor for AAD, an interesting finding of the registry was that 60% of patients had maximum aortic diameters <5.5 cm and 40% patients aortic diameters <5.0 cm.

**Figure 2.**
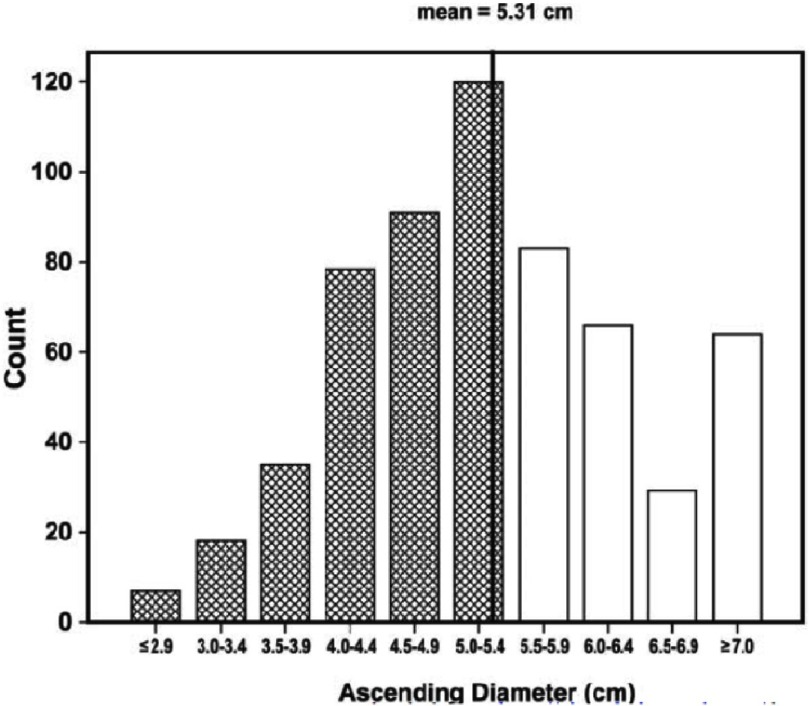
Distribution of aortic diameter (cm) at time of presentation with type A AAD. Shared bars indicate 59% of patients with diameter < 5.5 cm. Reproduced with permission of Pape LA et al. Circulation. 2007 Sep 4;116(10):1120-7.

Marfan syndrome patients were more likely to dissect at larger diameters (odds ratio, 14.3; p<0.002). Therefore, the current surgical guidelines for thoracic aortic aneurysm repair (<5.5 cm) would fail to prevent the majority of AAD seen in this cohort (Figure 2)^7^.

Similarly, in 21% of patients with type B AAD, aortic diameter was <35 mm^8,9^. A significantly higher frequency of AAD occurred from 6:00 a.m. to 12:00 noon than in other time periods. (p<0.001)^10^. The frequency of AAD was significantly higher in winter (p<0.008 versus other seasons), particularly in January (p<0.001)^11^.

## Presentation

Sudden onset of severe, sharp pain was the single most common presenting complaint. However, tearing, ripping or migratory were not common descriptors of pain in IRAD. Chest pain was significantly more common in patients with type A AAD (79% versus 63% of type B dissections), whereas back pain and abdominal pain were more common in type B AAD (64% and 43%, respectively)^1^.

Patients with abdominal pain tended to have delayed diagnosis and had experienced a higher mortality rate than those with more typical symptoms (28% vs 10%, p=0.02)^12^. Syncope was reported in 13%, often indicating a severe haemodynamic status, obstruction of cerebral vessels or stroke^13^. Pulse deficits were described in 30% and 20% of patients with a type A and type B dissection, respectively^14^. These patients had a higher rate of in-hospital complications^15^. Hypertension is the most common condition associated with AAD, although in time of presentation is more frequent in type B than in type A (70% vs 36 %). Patients with narrowed pulse pressure experienced more cardiac complications, particularly cardiac tamponade, whereas those with markedly elevated pulse pressure were more likely to have abdominal aortic involvement^16^.

**Figure 3.**
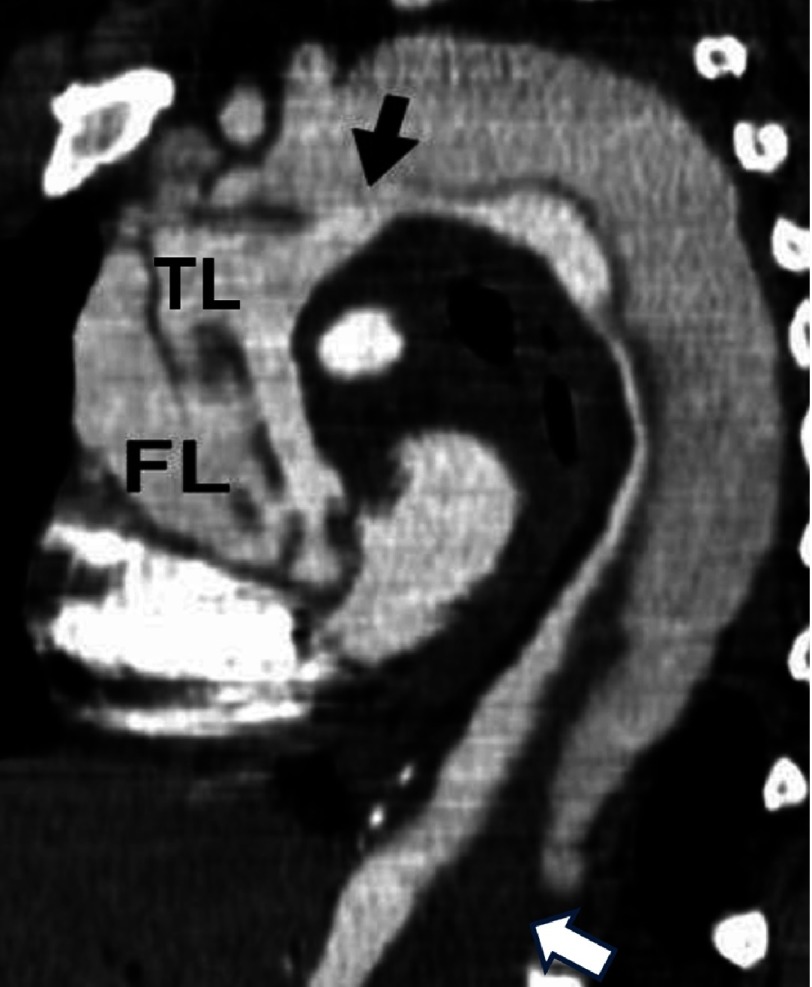
CT shows a type A AAD with a large entry tear in the aortic arch (black arrow) and retrograde dissection to the ascending aorta and antegrade descending thoracic aorta dissection with distal total thrombosis of false lumen (white arrow). Abbreviations: TL= true lumen, FL= false lumen.

## Diagnosis

Diagnosis of this disease requires a high degree of suspicion of an aortic dissection in patients who have some risk factors. Patients who present without pain are more likely to have a missed or delayed diagnosis^17^. Patients with atypical features such as fever or congestive heart failure required longer time intervals for diagnostic confirmation^18^. Transfer from an outside facility, fever and normal blood pressure were the greatest relative predictors of delay. In contrast, patients with hypotension, tamponade, signs of ischaemic lower limbs (as evidenced by pulse deficits), coma or altered consciousness were diagnosed earlier^15^. In the IRAD series, the ECG was normal in 31%, showed non-specific ST and T-wave changes in 42%, ischaemic changes in 15% and evidence of an acute MI in 5% of type A AAD. An abnormal ECG leads to delays in diagnosis^18^. Although typical radiographic findings of type A AAD include widening of the mediastinum or aortic knob, in fact >20% of patients with confirmed AAD lack abnormalities of the mediastinum or aortic contour^1^. The relatively non-specific finding of a pleural effusion was also seen in those with longer times to diagnosis^18^.

### Imaging studies

**Figure 4.**
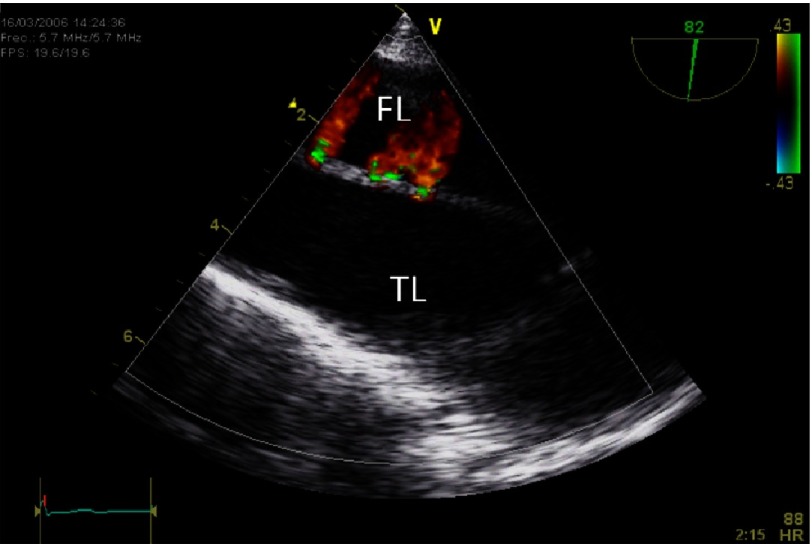
Transoesophageal echocardiography showing a type B AAD with secondary communications and small flows from true lumen (TL) to false lumen (FL).

**Figure 5.**
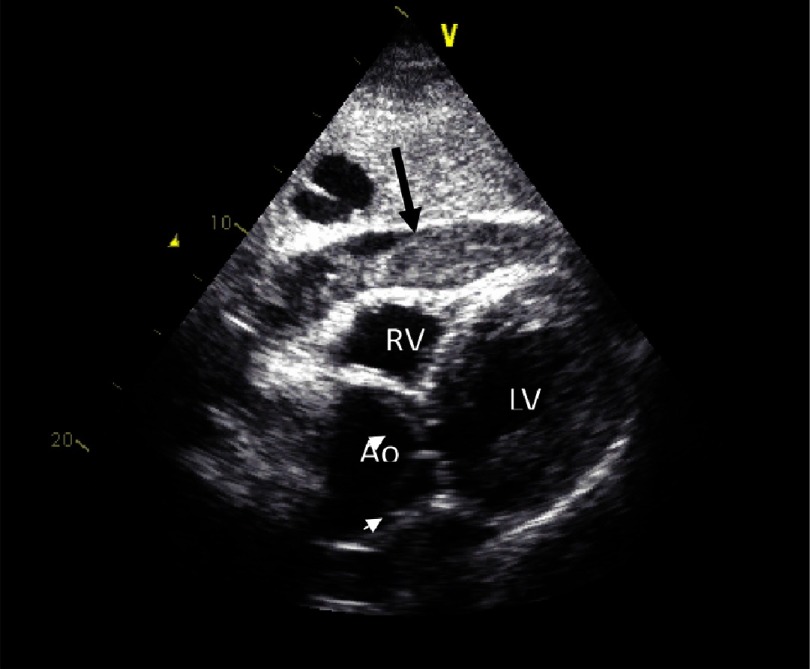
TTE shows pericardial effusion Black arrow) with compression of the right ventricle and collapse of the right atrium secondary to pericaldial tamponade. Note aortic root enlargement with an intimal flap (white small arrows).

Computed tomography (CT) was the initial imaging study in 69% of cases (Figure 3) echocardiography in 25%, magnetic resonance imaging (MRI) in 4% and aortography in 2-3%^19^. The use of transoesophageal echocardiography (TEE) as the initial diagnostic imaging study has decreased over time (Figure 4). One study demonstrated the value of the simple imaging information by TEE in the prediction of complications^20^. Evidence of pericardial effusion (p = 0.04), tamponade (p <0.01) (Figure 5), periaortic haematoma (p = 0.02), and patent false-lumen (p = 0.08) were more frequent in non-survivors^20,21^.

Use of imaging studies to confirm the diagnosis of AAD varied between North American and European centers. North American centers performed an average of 1.6 imaging studies compared with 1.8 in the European group (p = 0.002). Compared with Europeans, type A AAD had a substantial delay diagnosis in North Americans. However, no significant differences for early mortality rates were observed between the two groups^22^. Preoperative coronary arteriography was rarely performed in patients with AAD (11%). When performed, preoperative coronary arteriography was not associated with any significant changes in in-hospital and long-term mortality^23^.

### Biomarkers

D-dimer was markedly elevated in patients with acute aortic dissection^24^. Analysis according to control disease showed that the cutoff level of 500 ng/mL ruling out aortic dissection, had a negative likelihood ratio of 0.07 over the first 24 hours. However, lack of false-lumen patency or intramural haematoma may result in lower levels. D-dimer levels may be useful to rule out AAD particularly if used within the first 24 hours after symptom onset^25^.

The development of an evidence-based strategy to rule out aortic dissection in patients presenting chest pain would be useful given the high number of patients who attend emergency departments with chest pain. Rapid diagnosis of dissection is most likely when CT or echocardiography from part of the diagnostic testing. In contrast, when MRI or an aortogram were performed, the diagnosis was delayed, often significantly. In IRAD^18^, the use of MRI, which is not widely available particularly on an emergent basis, probably represents cases in which an alternative diagnosis was initially contemplated. Median time from arrival at the emergency room to diagnosis was 4.3 hours (quartile 1–3, 1.5–24 hours).

Delays in AAD occurred in female patients; those with atypical symptoms that were not sudden or did not include chest, back or any pain; patients with an absence of pulse deficit or hypotension; or those who initially attended to a nontertiary care hospital (all p<0.05). An interesting IRAD paper proved high sensitivity (>95%) of the clinical risk markers (risk conditions, symptom conditions and physical examination conditions) proposed in the 2010 thoracic aortic disease guidelines as a tool for the detection of AAD^26^.

## Management and in-hospital mortality

**Figure 6.**
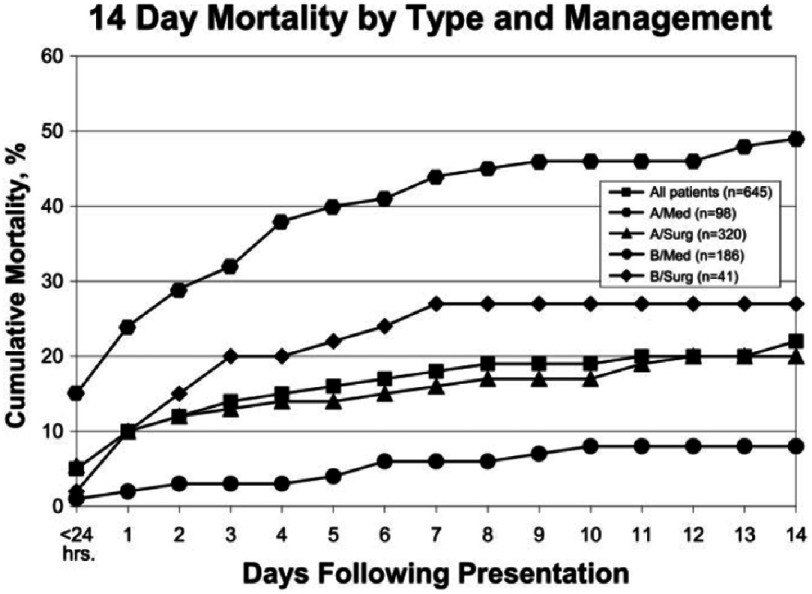
Acute phase mortality stratified by medical and surgical treatment in both type A and type B AAD. Reproduced with permission of Hagan PA. JAMA. 2000 Feb 16;283(7):897-903.

Management and mortality was significantly different for aortic dissection in IRAD depending on AAD was type A or type B and medical or surgical treatment (Figure 6). Overall mortality was 27.4%.

### Type A

The majority of patients presenting type A AAD were managed surgically (86%), with significantly more operative procedures undertaken in recent years compared to the late 1990’s (79% to 90%)^14^. Over time, the in-hospital mortality rate of patients presenting type A AAD dropped significantly from 31% to 22% mainly due to a decline in surgical mortality from 25% to 18%. The medical mortality rate among those with type A AAD remained high (57%) and did not change over time. The mean time interval from diagnosis to the surgical intervention was 4.3 hours (Q1-Q3, 2.4-14hours)^18^. Not surprisingly, the time period was shorter in unstable than in stable patients. Delay from diagnosis to surgery was associated with a history of previous heart surgery, and initial diagnosis at a nontertiary hospital (p<0.001)^18^.

### Type B

The majority of patients with type B AAD were treated medically (63%). In the last 20 years, this percentage has decreased (75% to 57%) as endovascular management has increased from 7% to 31%^14^. Open surgery decreased from 17% to 8%, although there was an increase in hybrid procedures that used surgical debranching techniques to facilitate endovascular intervention. The overall in-hospital mortality rate of patients presenting with type B did not change significantly (12% vs 14%).

One third of patients with acute type B dissection present with complications such as malperfusion syndromes, signs of imminent rupture, expansion, and/or haemodynamic instability. In these cases, classic open surgery or endovascular treatment is indicated. Overall mortality of open surgery was 30%, versus 10% when patients were treated with medical therapy alone^14^. The two independent predictors of surgical mortality of type B AAD were age >70 years (odds ratio, 4.32) and preoperative shock/hypotension (odds ratio, 6.05)^27^.

Endovascular treatment has emerged over the last decade. In-hospital mortality was significantly higher after open surgery (33.9%) than after endovascular treatment (10.6%, p=0.002)^28^. Therefore, in the IRAD, endovascular treatment appears to offer better short-term outcomes in terms of mortality and associated complications than open repair.

Ascending thoracic aortic enlargement in patients with acute type B AAD does not appear to predict an increased risk of mortality, but it is associated with more frequent open surgical intervention that often involves replacement of the proximal aorta. Those with smaller proximal aortas are more likely to receive endovascular therapy^29^. Isolated abdominal AAD is rare (1.3%) and had similar profile than the rest of type B AAD^30^. A new time classification of aotic dissection has been proposed by IRAD. When survival curves were constructed, 4 distinct time periods were noted: hyperacute (symptom onset to 24 hours), acute (2-7 days), subacute (8-30 days), and chronic (>30 days). Overall survival was progressively lower through the 4 time periods.

## Complications

AAD may cause some complications which have prognostic and management implications.

### Hypotension, shock and congestive heart failure

At hospital presentation, one of the most ominous findings on physical examination is hypotension (systolic blood pressure <90 mmHg). Hypotension occurred in >25% of patients with type A AAD and was associated with neurological deficits, altered mental status, myocardial and mesenteric ischaemia, limb ischaemia and death in 55% of patients^31^. Congestive heart failure on presentation was detected in 6.4% of patients and was associated with an increase in diagnosis delay given the atypical presentation^32^.

### Recurrent pain and refractary hypotension

Recurrent pain and refractory hypertension appeared as clinical signs associated with increased in-hospital mortality in patients with type B dissection (17.4% vs 4%), particularly when managed medically^33^. Multivariable analysis confirmed that presence of one theses two variables was a predictor of mortality, with an odds ratio of 3.3.

### Pericardial effusion and tamponade

Pericardial tamponade was detected in 18% of type A AAD^34^. Patients with pericardial tamponade were more likely to have periaortic haematomas (45% vs 21%, p <0.0001). In-hospital outcomes were significantly worse in patients with pericardial tamponade. Mortality of patients with pericardial tamponade remained significantly higher, even after adjustment for baseline clinical characteristics (p <0.001)^34^.

### Periaortic haematoma

Periaortic haematoma is a complication present in 23% of AAD and is more frequent in IMH than classical AD. A multivariate model showed periaortic haematomas to be an independent predictor of mortality in patients with aortic dissections (odds ratio 1.71, 95% confidence interval 1.15 to 2.54, p = 0.007^35^.

### Visceral and peripheral ischaemia

The mechanisms of visceral and peripheral ischaemia may be secondary to severe compression of the true lumen or secondary to branch vessel involvement. Patients with renal insufficiency are at increased risk for drug-resistant hypertension and aortic branch vessel compromise. Upon recognition, renal impairment indicates a need for close monitoring, aggressive blood pressure control, and evaluation of aortic branch vessel circulations^36^.

Type A acute aortic dissection complicated by mesenteric malperfusion is a rare (3.7%) but ominous complication carrying a high risk of hospital mortality^37^. In-hospital mortality of patients with mesenteric malperfusion receiving medical, endovascular and surgical/hybrid therapy was 95%, 73%, and 42%, respectively (p <0.001). Similarly, visceral ischemia was a complication in 7% of type B AAD and medical management was a predictor of mortality in multivariate analysis (OR, 5.91; p = 0.036)^38^.

Acute limb ischaemia was associated with acute renal failure (OR 2.7; p = 0.048), acute mesenteric ischaemia/infarction (OR = 6.9; p <0.001) and death (OR 3.5; p = 0.02).Endovascular, but not surgical therpy, was more commonly performed in patients with acute limb ischaemia (31% vs 10%, p = 0.004). Aortic fenestration or aorto-iliac stenting was the primary therapy in 93%; surgical bypass was used in 7%. Limb salvage was 93% at a mean of 18 months of follow-up^36^. Major brain injury at the onset of dissection was present in 10% of type A AAD. Surgery appeared to be associated with better early and late outcomes^39^. Brain injury reversal occurred in 80% of cases, and suggests that this injury, *per se*, should not contraindicate surgery, especially if patients do not present with signs of neurological devastation^40^.

## Predictors of mortality in acute phase

In-hospital mortality was highly dependent on patient risk profiles prior to surgery^41^. Patients classified as unstable (cardiac tamponade, shock, congestive heart failure, cerebrovascular acident, coma, myocardial infarction, acute renal failure, or mesenteric ischaemia) had much higher in-hospital mortality of 31.4% versus 16.7% in those without unstable features (p <0.001) (Table 3)^42^.

**Table 1.** Risk factors in patients with acute aortic dissection in IRAD.

Category	Total (% )	Type A	Type B	p
Hypertension	72	69	77	0.08
Atherosclerosis	31	24	42	<0.001
Known aortic aneurysm	16	12	2	0.006
Previous AAD	6	4	11	0.005
Diabetes mellitus	5	4	7	0.29
Previous cardiac surgery	18	16	21	0.16
Aortic valve replacement	5	6	5	0.65
Aortic aneurysm and/or AAD	10	7	14	0.02
Coronary artery bypass graft surgery	4	5	3	0.32
Marfan Syndrome	5	7	2	0.02
Iatrogenic	4	5	3	0.47

**Notes.**

Modified from Hagan P. JAMA. 2000 Feb 16;283(7):897-903.

**Table 2.** Independent Predictors of In-hospital Mortality in Type A AAD.

Variables at presentation	type A (% )	% among survivors	% among deaths	model P value	Mortality odds ratio (95% IC)
Age > 70 y	35.2	30.0	46.1	0.03	1.70 (1.05-2.77)
Female	34.5	30.7	42.7	0.20	1.38 (0.85-2.27)
Abrupt onset of pain	84.5	82.3	89.0	0.01	2.60 (1.08-3.52)
Abnormal ECG	69.6	65.2	79.5	0.03	1.77 (1.06-2.95)
Any pulse deficit	30.1	24.7	41.1	0.004	2.03 (1.25-3.29)
Kidney failure	5.6	2.9	11.9	0.002	4.77 (1.80-12.6)
Hypotension/shock/tamponade	29.0	20.1	47.1	<0.0001	2.97 (1.83-4.81)

**Notes.**

Modified from Trimarchi et al. Circulation. 2002 Aug 27;106(9):1110-5.

**Table 3.** Independent Predictors of In-Hospital Mortality in Type B Aortic Dissection.

Variables at Presentarion	Mortality Odds Ratio (95% IC)	Parameter Coefficient	Model Score Assigned	P Value
Female	1.37 (0.67-2.81)	0.316	0.3	0.387
Age (per decade)	1.33 (1.00-1.75)	0.28	0.3	0.044
Hypotension/shock	6.43 (2.18-18.98)	1.861	1.9	0.001
Periaortic hematoma	3.06 (1.38-6.78)	1.119	1.1	0.006
Diameter ≥5.5cm	3.06 (2.87-12.73)	1.798	1.8	<0.001
Mesenteric ischemia	9.03 (3.49-23.38)	2.201	2.2	<0.001
Acute renal failure	3.61 (1.68-7.75)	1.284	1.3	0.001
Limb ischemia	3.02 (1.05-8.68)	1.105	1.1	0.040

**Notes.**

Reproduced with permission of Tolenaar JL. Circulation. 2014 Sep 9;130(11 Suppl 1):S45-50.

Independent pre-operative predictors of operative mortality were; a history of aortic valve replacement (OR 3.12; p=0.02), migrating chest pain (OR 2.77; p=0.001), presenting hypotension (OR 1.95; p=0.02), presenting shock or cardiac tamponade (OR 2.69; p=0.002), and pre-operative limb ischaemia (OR 2.10; p=0.04)^42^.

Three key variables, collectively named “the deadly triad”, emerged as highly significant predictors of death: hypotension/shock (odds ratio (OR 23.8, p <0.001), absence of chest/back pain on presentation (OR 3.5, p<0.01), and branch vessel involvement (OR 2.9, p<0.02)^43^. Independent preoperative predictors of mortality in model 1 were; age greater than 70 years, prior cardiac surgery, hypotension (systolic blood pressure less than 100 mm Hg) or shock at presentation, migrating pain, cardiac tamponade, any pulse deficit, and electrocardiogram with findings of myocardial ischemia or infarction.

In model 2, other predictors of surgical death were intraoperative hypotension, a right ventricle dysfunction at surgery, and a necessity to perform coronary revascularization^44^. An independent predictor for favorable surgical outcome was right hemiarch replacement. In patients more-extensive roor replacement interventions were not associated with increased hospital mortality. This supports such an approach in young patients and patients with connective tissue diseases and bicuspid aortic valves^45^.

In type B AAD, predictors for in-hospital mortality were increasing age (OR 1.03; p=0.044), hypotension/shock (OR 6.43; p=0.001), periaortic hematoma (OR 3.06; p=0.006), descending diameter ≥5.5 cm (OR 6.04; p<0.001), mesenteric ischemia (OR 9.03; p<0.001), acute renal failure (OR 3.61; p=0.001), and limb ischemia (OR 3.02; p=0.040). Based on these multivariable results, a reliable and simple bedside risk prediction tool was developed (Table 4)^46^.

## Long-term outcome

Contemporary 1- and 3-year survivals in patients with type A AAD treated surgically are excellent. Survival for type A patients treated with surgery was 96.1% and 90.5% at 1 and 3 years. Independent predictors of survival during the follow-up period do not appear to be influenced by in-hospital risks but rather by pre-existing comorbidities^47^. In AAD type B patients, three-year survival for patients treated medically, surgically or with endovascular therapy was 77.6%, 82.8%, and 76.2%, respectively (median follow-up 2.3 years, log-rank p=0.61).

Independent predictors of follow-up mortality included female sex (hazard ratio [HR], 1.99; p=0.03), a history of previous aortic aneurysm (HR, 2.17; p=0.04), a history of atherosclerosis (HR, 2.48; p=0.01), in-hospital renal failure (HR, 2.55; p=0.02), pleural effusion on chest X-ray (HR, 2.56; p=0.02), and in-hospital hypotension/shock (HR, 12.5; p=0.01)^48^.

**Figure 7.**
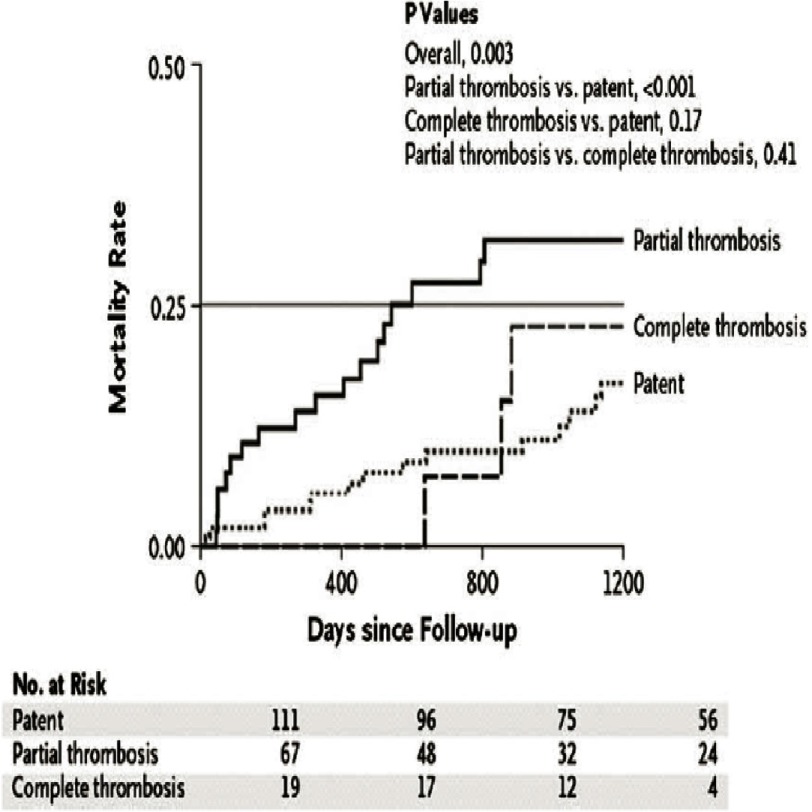
Kaplan-Meyer mortality curve stratified according to the status of the false lumen. P values were calculated by the long-eank test. Overall comparison of all three curves. Reproduced with permission from N Engl J Med. 2007 Jul 26;357(4):349-59.

Three main management issues are predominant during the follow-up phase and include: a) medical therapy; b) serial imaging to detect aortic complications (dissection progression, redissection or aneurysm formation); and c) reoperation when indicated. Multiple factors such as advanced age, aorta size and false-lumen flow may identify a higher-risk cohort for complications. Aortic diameter has long been the mainstay of aortic dissection prevention in patients with aortic aneurysm and of decisions to operate on remaining dissecting aneurysms in those recovering from acute type A or B. In addition to aortic diameter, patency of the false-lumen has been linked to false-lumen expansion and rupture. IRAD evaluated the impact of partial false-lumen thrombosis on long-term outcomes in patients with chronic type B aortic dissections^49^. Partial thrombosis of the false-lumen was present in 33.8% of acute type B aortic dissection on imaging and was a significant independent predictor of death at 3 years (RR, 2.69; p=0.002) showing 3-year mortality of 31.6% (Figure 7).

Multivariate models also showed the use of β blockers to be associated with improved survival in patients with type A undergoing surgery (odds ratio 0.47; p = 0.02) and the use of calcium channel blockers was associated with improved survival in type-B-patients treated medically (odds ratio 0.55; p = 0.01). The use of angiotensin-converting enzyme inhibitors showed no association with mortality^50^. In addition, the use of calcium-channel blockers after type B AAD may reduce the rate of aortic expansion^51^.

## Intramural haematoma

**Figure 8.**
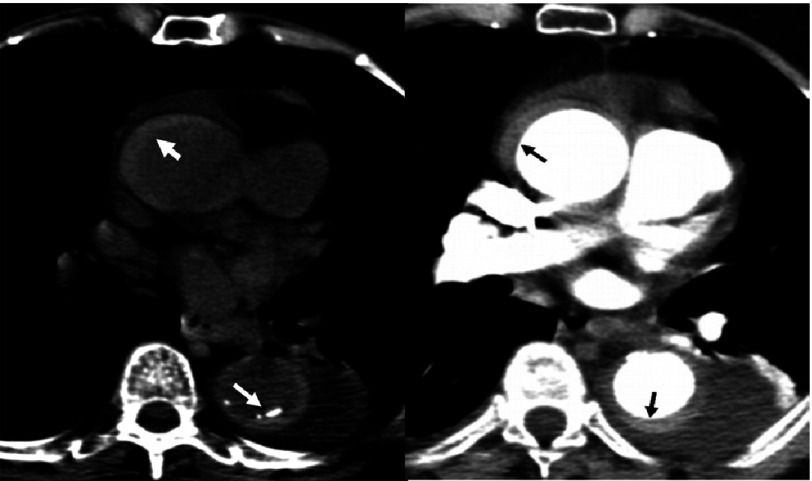
Intramural haematoma. Left) non-enhanced CT showing a high attenuation signal in the aortic wall of the ascending and descending aorta (white arrows); right) CT angiography showing the same intramural haematoma but with more difficulties (black arrows).

Intramural haematoma (IMH) is a major type of aortic dissection. In the IRAD registry, 7% of patients had IMH^52^. This cohort tended to be older (68.7 versus 61.7 years; p <0.001) and more likely to have distal aortic involvement (60.3% versus 35.3%; p <0.0001). Type A IMH were less likely to present aortic regurgitation or pulse deficit, but more frequently periaortic haematoma or pericardial effusion than ADD. (Figure 8). Like classic AD, IMH is a highly lethal condition when it involves the ascending aorta (26.5% vs 26,5%). Type A IMH managed medically has significant mortality (40%) although less than classical dissection (62%). The investigators demonstrated an association between increasing hospital mortality and the proximity of IMH to the aortic valve, regardless of medical or surgical treatment^53^. Patients with type B IMH more often had periaortic hematoma (22% vs 13%; p = 0.020) and were more often treated medically (88% vs 62%; P <.001) 60. However, in these patients hospital mortality was lower than classical dissection (4.4% vs 11%). Fully 16% of patients have evidence of evolution to dissection on serial imaging.

## Conclusions

In the 20 years since the IRAD was established surgical mortality in patients with type A AAD has decreased significantly and endovascular treatment has emerged as an excellent option in complicated type B dissection. Improvements in AAD outcomes are likely due to a number of factors: earlier diagnosis, advances in surgical and endovascular techniques, and better perioperative management. In the future, regional networks will permit the most rapid diagnosis and transfer of patients with AAD to aortic centres of excellence for definitive treatment.
